# Supplementary material for: Patterns of Intron Gain and Loss in Fungi
Source: PLoS Biol. 2004 Nov 30;2(12):e422. doi: 10.1371/journal.pbio.0020422 (PMC532390; doi:10.1371/journal.pbio.0020422)
Supplement: Table S1 — Also available at http://genes.mit.edu/NielsenEtAl/. (4.3 MB ZIP). [file pbio.0020422.st001.zip › NielsenEtAl/html/421.html]

AN8785.1.NCU01241.1.MG07066.1.FG07131.1


```
 CLUSTAL W (1.82) Multiple Sequence Alignments - Introns Inserted


Sequence 1: NCU01241.1	706 aa
Sequence 2: MG07066.1	689 aa
Sequence 3: FG07131.1	695 aa
Sequence 4: AN8785.1	707 aa
Alignment Length: 726 aa
Number Identitical Residues: 384 aa
Alignment Score (without introns) 17836


MG07066.1 	MAAAKASAVKEAVKETLIGS----TEEPAKLSAQTKARFTKYAVSDA-SGELAMGPEEFI
NCU01241.1	--MSTAAVVKEAVKETLIGSDEHAAADQVQPTAQTKARFTHYAVQDSETGELFLGPDQFI
FG07131.1 	--MSKLAVATEAVKEAVVGT-----HEPEQLAAHTKARFIQYAVKDSETGELYLGPEEFI
AN8785.1  	---MTMTGVKEAVKESLVGT----TVDEPSLSKQVKANFIKHARKDEATGELYMTEADFV
          	    . : ..*****:::*:    : :  . : :.**.* ::* .*  :*** :   :*:

MG07066.1 	NAVAPAEEDY0HKIQREQYSILFRVADRKGSGKVTLSDWAYFENILSKPDAEYEIAFRLF
NCU01241.1	DAVAPPHEDY0HKIKREQYSILFSVADRANKGKVTLSDWTYFENLLEKPDAEYEIAFRLF
FG07131.1 	NAVAPTGEDY0HKISRDQYSILFSVADVNGKGRVSLGDWGVFEHLLSKPDAEYQIAFRLF
AN8785.1  	DAIAPKHEDY0HKIKREHYGILFRVADSRRTGRINIHEWATFENLLAKPDAEYEIAFRLF
          	:*:**  *** ***.*::*.*** ***   .*::.: :*  **::* ******:******

MG07066.1 	DVERLGTVKYEDFRRLYELNKGEHTIPFNWDCEWAKLYIGDKKKRHNMDYQQFSQMLRGL
NCU01241.1	DVERLGTVKYDDFRRLYELNKGPNSIPFDWDCEWAKLYIGSKTKRHNLDYQQFSQMLRGL
FG07131.1 	DVERTGAVKYDDFRTLYEMNKGPDNLTFDWESDWAKIYIGNRKHRHPLDYPQFCQMLRGL
AN8785.1  	DTDGTGTVKWETFQKLYNENKTKDSIPFDWNSEWAALYTGKKKTRHDMTYPQFAQMLRGL
          	*.:  *:**:: *: **: **  ..:.*:*:.:** :* *.:. ** : * **.******

MG07066.1 	QGERIRQAFQIFDKDGDGYIEPEEFERIILETSKHKLSDHVLLHLGTLCNISQGSKVSYA
NCU01241.1	QGERIRQAFQLFDKDGDGFIEPGDFERIIMETSKHKLSDHLLENLNTLCNISQGSKVSYA
FG07131.1 	QGERIRQAFHQLDKDGDGFISGEEFERIIVETARHKLSEHILDNLHTLVNLSIGSKISYA
AN8785.1  	QGERIRQAFHVFDKDGDGYIEPEEFQRIILETSRHKLSDYVLEHLPSLCNISAGTKISYA
          	*********: :******:*.  :*:***:**::****:::* :* :* *:* *:*:***

MG07066.1 	NVRAFQNMIKEMDLVELI0A---------------------AKITRFSLFTPMEADILFH
NCU01241.1	NVRAFQNIIKEMDLVELI~IRKACGKSQDGKITRTEFLNQAAKITRFSLFTPMEADILFH
FG07131.1 	NVRAFQNVIGGMDLVEII~LRRAIDQSKDGKITRPEFLNEAAKLTRFSLFTPMETDILFH
AN8785.1  	TVRAFQNVMREMDMIDLI~IREATDKSEDGKITRSDFLNAAARLTRFSLFTPMEADILFH
          	.******::  **::::*    : ..:.... : ..  . :*::**********:*****

MG07066.1 	FASLDEPSGRLGLRDFAKVLDPSWRNLDSDDLAPKLAAA----VGQSTASKFLGNAMESA
NCU01241.1	FASVGNPSGRLGLEDFAKVLDPSWRNR--HEAEARLLPQ----AVQTKGQQFAHRAGESA
FG07131.1 	FAGLDEPSGRLGLSDFAKVLDPSWRNPIYDAVEATKAKV----ADGG----IMMSVLTSS
AN8785.1  	FAGLDAPSGRLSQKDFSKVIDASWRIPVAAAEQAITTASGAAHKAADAGKSMLHGVLESV
          	**.:. *****.  **:**:*.***        .     .::      ...:   .  * 

MG07066.1 	YNFALGSVAGAFGAFMVYPIDLVKTRMQNQRGVRPGERLYKNSIDCFQKVVRNEGFLGLY
NCU01241.1	YNFILGSLSGAFGAFMVYPIDLVKTRMQNQRGASPGSRLYDNSIDCFRKVIRNEGFRGLY
FG07131.1 	YNFALGSVAGAFGAFMVYPIDLVKTRLQNQRGAQPGQRLYKNSIDCFQKVIRNEGFRGLY
AN8785.1  	HHFALGSLAGAFGAFMVYPIDLVKTRMQNQRSSRVGERLYNNSIDCARKVIRNEGFTGLY
          	::* ***::*****************:****.   *.***.***** :**:***** ***

MG07066.1 	SGVLPQLVGVAPEKAIKLTVNDLVRGWTTDK-NGKIGLPSEILAGGTAGACQV0VFTNPL
NCU01241.1	SGVLPQLVGVAPEKAIKLTVNDLVRGAFTDK-QGNISVIHEIIAGGTAGGCQV0VFTNPL
FG07131.1 	SGVLPQLVGVAPEKAIKLTVNDIARKAFTDK-NGNITLWSEMVSGGSAGACQV0VFTNPL
AN8785.1  	SGVIPQLIGVAPEKAIKLTVNDLVRGYFAGKQNGKLKTWQEVLAGGSAGACQV0VFTNPL
          	***:***:**************:.*   :.*.:*::    *:::**:**.*** ******

MG07066.1 	EIVKIRLQVQGEVAK--TVEG-APKRSAMWIVRNLGLVGLYKGASACLLRDVPFSAIYFP
NCU01241.1	EIVKIRLQVQGEVAK--SVEG-APKRSAMWIVRNLGLVGLYKGASACLLRDVPFSAIYFP
FG07131.1 	EIVKIRLQVQGEVAK--TVEG-TPKRSAMWIVRNLGLVGLYKGASACLLRDVPFSAIYFP
AN8785.1  	EIVKIRLQVQGEIAKNAGVEGAAPRRSALWIVRNLGLVGLYKGASACLLRDVPFSAIYFP
          	************:**.: ***::*:***:*******************************

MG07066.1 	AYSHLKKDVFGESPTKKLGVLQLLLSGAIAGMPAAYLTTPFDVIKTRLQVEQRKGETSYT
NCU01241.1	TYSHLKKDLFGESKTKKLGVLQLLTAGAIAGMPAAYLTTPCDVIKTRLQVEARKGDTQYT
FG07131.1 	TYSHLKKDFFGESPTNKLGVLQLLTAGAIAGMPAAYLTTPCDVIKTRLQVEARKGEATYN
AN8785.1  	TYSHLKSDFFGESRTHKLGVVQLLTAGAIAGMPAAYLTTPCDVIKTRLQVEARKGEKAYT
          	:*****.*.**** *:****:*** :************** ********** ***:  *.

MG07066.1 	GLRHAATTIMKEEGPRAFFKGGLARIFRSSPQFGFTLTAYEILQTAIPYPGSKSASQDQL
NCU01241.1	GLRHAAKTIWKEEGFRAFFKGGPARIFRSSPQFGFTLAAYELLQSVLPYPGQK---AEKM
FG07131.1 	GLRHAAKTIWKEEGLTAFFKGGPARIFRSSPQFGFTLAAYEVLQTVLPMPGTQ---KEKI
AN8785.1  	GLRHAAVTIFRDEGFKAFFKGGPARILRSSPQFGFTLAAYEVLQKWLPMPGSE---HDVS
          	****** ** ::**  ****** ***:**********:***:**. :* ** :    :  

MG07066.1 	HPGIAGGSGITTVATLSEKKADSTPFARSRNALKIILDLDEDFGRMKR-PDEEAWKSLPA
NCU01241.1	HAGVAD-----AVSMVKDSVGGDSPYTRSRNALKILLDLDEHFGRVKLDANAAGWKALPT
FG07131.1 	PTGVSD-----ALSTAKGS-LDTSPYGRSRNALKVILDLDEDFGKVKL-PNEKGWRSLPK
AN8785.1  	PTGQVEPG--VGVPVAKEP----LPYLRSRNALKLILDLDENIGRLPI-PDKERWPKFMR
          	 .*    .    :.  .       *: *******::*****.:*::   .:   *  :  

MG07066.1 	VMGGKSN--
NCU01241.1	WMQTPPKTA
FG07131.1 	IMGGGGQ--
AN8785.1  	PAAGAEK--
          	      :
```
